# Comparison of visual biofeedback system with a guiding waveform and abdomen-chest motion self-control system for respiratory motion management

**DOI:** 10.1093/jrr/rrv106

**Published:** 2016-08-03

**Authors:** Yujiro Nakajima, Noriyuki Kadoya, Takayuki Kanai, Kengo Ito, Kiyokazu Sato, Suguru Dobashi, Takaya Yamamoto, Yojiro Ishikawa, Haruo Matsushita, Ken Takeda, Keiichi Jingu

**Affiliations:** 1 Department of Radiation Oncology, Tohoku University Graduate School of Medicine, 1-1 Seiryo-machi, Aoba-ku, Sendai, 980-8574, Japan; 2 Department of Radiology, Tohoku University Hospital, 1-1 Seiryo-machi, Aoba-ku, Sendai, 980-8574, Japan; 3 Department of Radiological Technology, Health Sciences, Tohoku University, Graduate School of Medicine, 1-1 Seiryo-machi, Aoba-ku, Sendai, 980-8574, Japan

**Keywords:** radiotherapy, four-dimensional CT, respiratory motion management, visual biofeedback, breathing guidance

## Abstract

Irregular breathing can influence the outcome of 4D computed tomography imaging and cause artifacts. Visual biofeedback systems associated with a patient-specific guiding waveform are known to reduce respiratory irregularities. In Japan, abdomen and chest motion self-control devices (Abches) (representing simpler visual coaching techniques without a guiding waveform) are used instead; however, no studies have compared these two systems to date. Here, we evaluate the effectiveness of respiratory coaching in reducing respiratory irregularities by comparing two respiratory management systems. We collected data from 11 healthy volunteers. Bar and wave models were used as visual biofeedback systems. Abches consisted of a respiratory indicator indicating the end of each expiration and inspiration motion. Respiratory variations were quantified as root mean squared error (RMSE) of displacement and period of breathing cycles. All coaching techniques improved respiratory variation, compared with free-breathing. Displacement RMSEs were 1.43 ± 0.84, 1.22 ± 1.13, 1.21 ± 0.86 and 0.98 ± 0.47 mm for free-breathing, Abches, bar model and wave model, respectively. Period RMSEs were 0.48 ± 0.42, 0.33 ± 0.31, 0.23 ± 0.18 and 0.17 ± 0.05 s for free-breathing, Abches, bar model and wave model, respectively. The average reduction in displacement and period RMSE compared with the wave model were 27% and 47%, respectively. For variation in both displacement and period, wave model was superior to the other techniques. Our results showed that visual biofeedback combined with a wave model could potentially provide clinical benefits in respiratory management, although all techniques were able to reduce respiratory irregularities.

## INTRODUCTION

Respiratory motion affects the accuracy of radiotherapy in the processes of imaging and radiation delivery. If respiratory motion is not accounted for during image acquisitions, including 4D computed tomography (4D-CT) and 4D positron emission tomography (4D-PET), this motion causes artifacts in the images, such as blurring and overlapping of structures [1–3]. These artifacts can influence the target/normal surrounding tissue delineation and a misestimate of the organ motion during treatment planning. In addition, respiratory motion can affect the efficiency and accuracy of radiation delivery, including respiratory-gated radiotherapy, real-time tumor-tracking radiotherapy and dynamic tumor tracking radiotherapy. These deleterious effects of respiratory motion lead to the radiation-induced normal tissue complication.

Many investigators have reported on respiratory motion management techniques and their effectiveness to address the problem of respiratory irregularities [4–11]. George *et al*. proposed an audiovisual biofeedback system, without a guiding waveform, able to significantly reduce residual motion variability for a given duty cycle during respiratory-gated radiotherapy [4]. Audiovisual biofeedback systems including a patient-specific guiding waveform, developed by Venkat *et al*., have been shown to significantly reduce cycle-to-cycle variation in displacement and period compared with free breathing [5]. On the other hand, the Abches system (APEX Medical Inc., Tokyo), an abdomen and chest motion self-control device representing a simpler visual coaching technique without a guiding waveform, is the main method used in Japan [9]. Tarohda *et al*. demonstrated that Abches can achieve respiratory control with high accuracy and reproducibility for inoperable non–small cell lung cancers [8]. Set against this background, no studies have comparatively evaluated these different respiratory motion management techniques to date. The aim of this study was to assess the effectiveness of respiratory coaching to reduce respiratory irregularities using two respiratory motion management systems.

## MATERIALS AND METHODS

### Patient characteristics

A total of 11 healthy volunteers with a median age of 23 years (22–33) participated in this study. All participants provided informed written consent to be included in this study.

### Visual biofeedback system

**Fig. 1.**
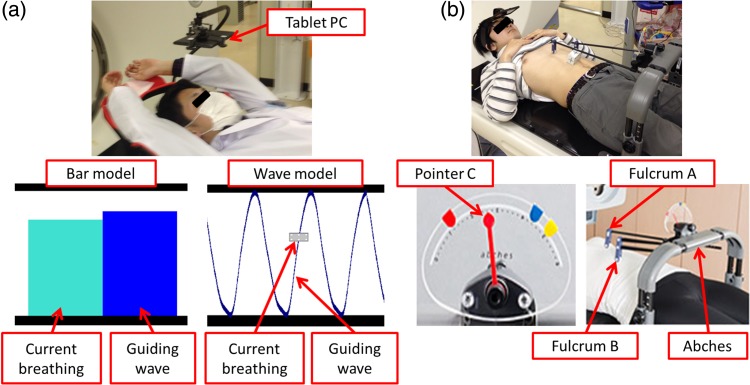
(**a**) Audiovisual biofeedback system. Bar or wave models are displayed on a tablet PC. (**b**) Abches system.

We used the visual biofeedback system developed by Stanford and Sydney Universities and as described by Venkat *et al*. [5]. This system includes two visual feedback models: a bar and a wave model (Fig. 1a). To determine the guiding waveform, we collected data from ten individual breathing cycles. These cycles were determined on the basis of real-time phase estimation using a Real-time Positioning Management (RPM) system (Varian Medical systems, Palo Alto, CA). The guiding breathing cycle was estimated by averaging the coefficients of finite Fourier series expansions for each of the individual breathing cycles. This step allowed elimination of any free breathing cycles deemed inappropriate. We used a tablet PC (Nexus 7, Google, CA) and frame to display the guiding wave.

### Abdomen and chest motion self-control device

The respiration-monitoring system, Abches, was developed by Onishi at the University of Yamanashi, Japan. The Abches system consists of a main unit made of plastic and a minimum amount of metal. To use this system, Fulcrum A and B are placed on the patient's abdomen and chest, respectively. During respiration, the device pointer C moves, along with the fulcrum (Fig. 1b), serving as an external device to monitor respiration. Further details on the Abches system have been provided elsewhere [8].

### Qualification of respiratory irregularity

**Fig. 2.**
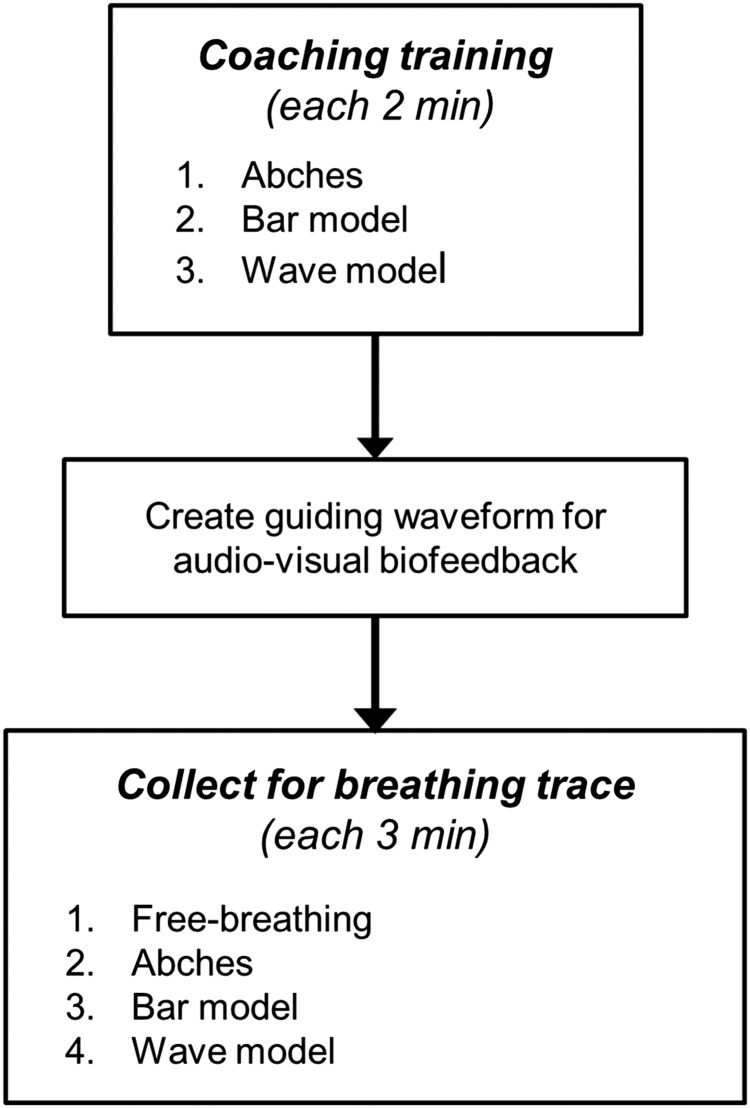
Breathing trace acquisition flowchart.

Figure 2 summarizes the data acquisition process, including the following:

- Two minutes of experimental coaching training for Abches, bar and wave models.
- Creation of a guiding wave for visual biofeedback.
- Collecting of breathing trace data for free-breathing, followed by Abches, bar and wave models during 3-min intervals.

To prevent experimental bias, the order in which the coaching training techniques were introduced varied among volunteers.

We calculated the root mean squared error (RMSE) for displacement and period of breathing cycles to quantitatively estimate respiratory variations. Displacement RMSE was calculated for each waveform and compared with the average waveform in phase domain (Eq. 1):

$$\text{Displacement RMSE}=\frac{\sum_{All\;Cycles}\sqrt{\sum_{i=1}^{360}\frac{{\left(x_{i}-y_{i}\right)}^{2}}{360}}}{Total\;Cycles},$$

where $x_{i}$ is the displacement measured during each cycle waveform, $y_{i}$ is the average waveform at phase *i*, and the analysis is performed for every degree of phase (hence 360). Period RMSE was also computed for each waveform.

### Statistical analyses

Statistical analyses were performed to quantify the differences between the two distinct respiratory management systems. We tested whether the evaluation metrics used for each respiratory management differed significantly from free-breathing (*P* < 0.05) using a paired *t*-test in JMP Pro 11.0.0 (SAS Institute Inc., Cary, NC).

## RESULTS

**Fig. 3.**
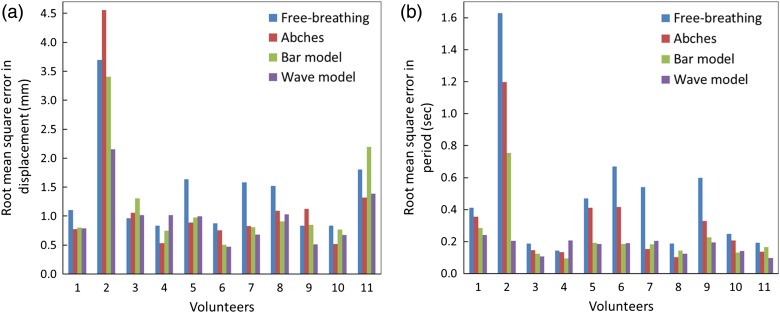
(**a**) Root mean square error for the displacement for each volunteer and the four types of training: free-breathing, Abches, bar model and wave model. (**b**) Root mean square error for the period for each volunteer and the four types of training: free-breathing, Abches, bar model and wave model.

All coaching techniques were able to improve respiratory variation, compared with free-breathing. Displacement RMSE values for all volunteers are shown in Fig. 3a. Displacement RMSE decreased in 8 of 11 cases, 9 of 11 cases, and 9 of 11 cases for Abches, bar and wave models, respectively. Period RMSE values for all volunteers are shown in Fig. 3b. Period RMSE decreased in 11 of 11 cases, 11 of 11 cases, and 10 of 11 cases for Abches, bar and wave models, respectively.

**Fig. 4.**
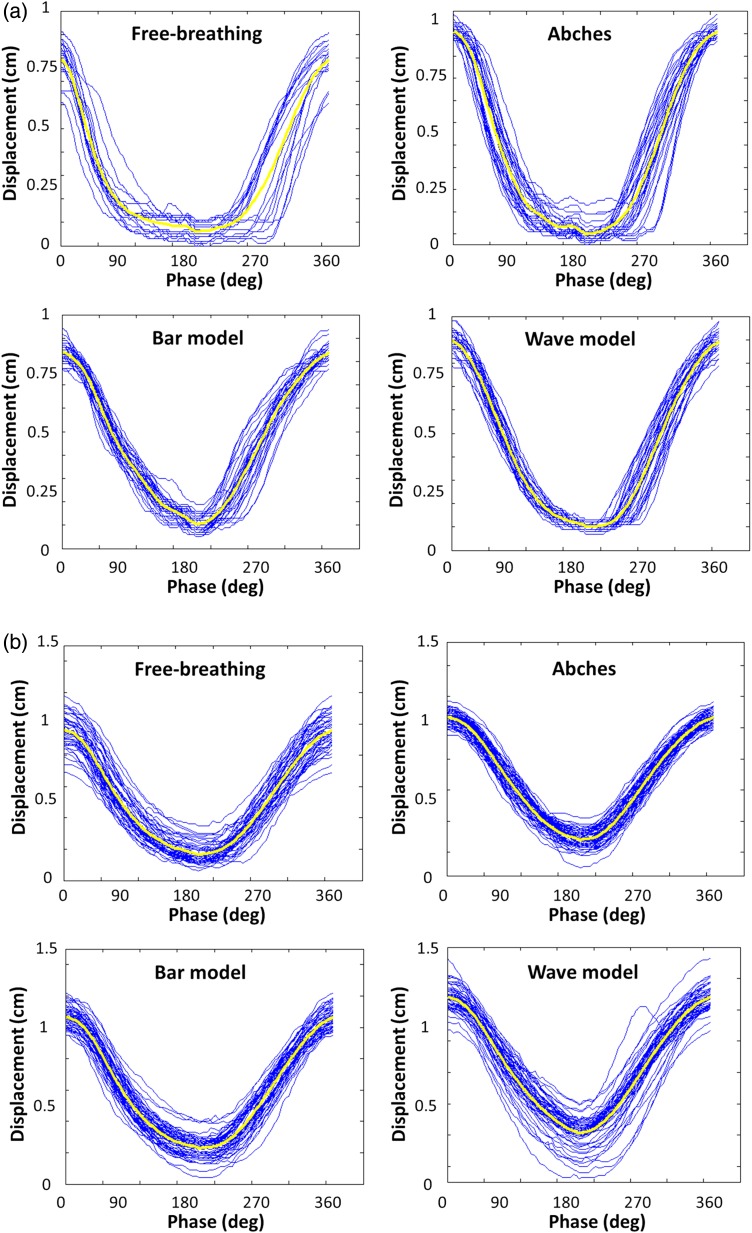
(**a**) Example of a respiratory trace of Volunteer 6 with individual breathing cycle (blue line) and average waveform (yellow line). The respiratory variation was improved using coaching techniques. (**b**) The respiratory trace for the wave model of Volunteer 4 using individual breathing cycle and average waveform was not improved.

Coaching techniques were shown to be effective in reducing respiratory variation in most volunteers (Fig. 4a); however, the traces of some of the volunteers did not improve with respect to respiratory irregularity. For example, the displacement and period RMSE decreased in Volunteer 4 if Abches or the bar model were used, but did not improve using the wave model (Fig. 4b). This is possibly due to the higher complexity of the wave model compared with other coaching techniques. Therefore, increasing training time above the 2 min used in this experiment could improve the outcome of the wave model.

Table 1 shows a displacement RMSE average of 1.22 ± 0.84 (Abches) and 0.98 ± 0.47 (wave model) for all volunteers, demonstrating the better performance of the visual biofeedback system in reducing displacement variation compared with Abches (−18% vs −27%). We found significant differences only between free-breathing and the wave model (*P* < 0.05). Table 2 shows the period RMSE values averaged over all volunteers, highlighting that the bar and wave models were more efficient at reducing period variation than Abches (−45% and −47 vs −30%). We found significant differences only between free-breathing and all coaching techniques (*P* < 0.05). The wave model was superior to free-breathing, the bar model, and Abches with respect to period and displacement variation.

**Table 1.** Displacement RMSE values averaged over all volunteers for each coaching technique, for free-breathing, and in relation to the reduction in displacement RMSE values associated with each coaching technique (average ± SD)

Coaching technique	Displacement RMSE (mm)	Displacement RMSE reduction (%)	*P* value
Free-breathing	1.43 ± 0.84	–	–
Abches	1.22 ± 1.13	−18 ± 28	0.19
Bar model	1.21 ± 0.86	−15 ± 28	0.09
Wave model	0.98 ± 0.47	−27 ± 23	<0.05

**Table 2.** Period RMSE values averaged over all volunteers for each coaching technique, for free-breathing, in relation to the reduction in displacement RMSE values associated with each coaching technique (average ± SD)

Coaching technique	Period RMSE (s)	Period RMSE reduction (%)	*P* value
Free-breathing	0.48 ± 0.42	–	–
Abches	0.33 ± 0.31	−30 ± 19	<0.05
Bar model	0.23 ± 0.18	−45 ± 19	<0.05
Wave model	0.17 ± 0.05	−47 ± 34	<0.05

RMSE = root mean squared error.

## DISCUSSION

We have demonstrated the effectiveness of respiratory coaching at reducing respiratory irregularities using two respiratory motion management systems. These results are consistent with those previously reported, suggesting that breathing coaching can reduce respiratory variations [4, 5]. Venkat *et al*. reported a reduction in displacement RMSE values of 55% and a reduction in period RMSE values of 75% for visual biofeedback combined with a wave model. In contrast, our results using the wave model showed a reduction in displacement and period RMSE values of 27% and 47%, respectively [5]. The reason for a smaller reduction in respiratory variation in our study compared with in previous reports could be that volunteers might not fully understand due to a shorter coaching training time.

Visual biofeedback using the wave model was superior to Abches, for both displacement and period RMSE, because Abches guides the inhale–exhale–inhale transition, and only extreme values are displayed. On the other hand, the wave model could enable a future trend of anticipating motion. Furthermore, visual biofeedback was more sensitive to respiratory motion than Abches. As a consequence, displacement and period RMSE values could be further improved using bar and wave models compared with Abches.

There are several limitations of reproducibility in this study. First, the data presented here was collected from healthy volunteers, rather than from lung cancer patients usually investigated in this type of research. These results may be applicable to some cancer patients with good lung function, such as pancreas and liver cancer patients. Our results could also be useful in clinical settings for lung cancer patients with compromised pulmonary function, where intensive training before 4DCT is implemented. Second, this study collected breathing trace data in only one session. Thus, our result might cause the lower reproducibility. Further study should be conducted with larger sample sizes and more repeat sessions for each patient. This study demonstrated the use of respiratory coaching in reducing respiratory irregularities. In particular, visual biofeedback combined with a wave model was found to be useful in reducing respiratory motion variation, thus reducing the deleterious effects of respiratory motion (reduced accuracy of radiotherapy in the processes of imaging and radiation delivery).

## CONCLUSION

In this study, we evaluated the efficacy of visual biofeedback in reducing respiratory irregularities compared with Abches. Our results showed that visual biofeedback combined with a wave model was potentially able to provide clinical benefits during respiratory management, although all techniques were proven efficient in reducing respiratory irregularities.

## FUNDING

This work was supported by a Japan Society for the Promotion of Science Grant-in-Aid for Young Scientists (B) (JSPS 15K19765) and a Research Grant from the Japan Radiological Society (funded by Bayer).
